# Inhibition of neutral sphingomyelinases in skeletal muscle attenuates fatty-acid induced defects in metabolism and stress

**DOI:** 10.1186/2193-1801-3-255

**Published:** 2014-05-20

**Authors:** Mahesh Kumar Verma, Aggunda Nagaraju Yateesh, Korrapati Neelima, Niketa Pawar, Kandoor Sandhya, Jayaram Poornima, Mudigere N Lakshmi, Sivakumaran Yogeshwari, Puttrevana M Pallavi, Anup M Oommen, Baggavalli P Somesh, Madanahalli R Jagannath

**Affiliations:** Connexios Life Sciences Private Ltd., No. 49, First Main road, 3rd phase, JP Nagar, Bangalore, 560 078 India

**Keywords:** Sphingomyelinase, Ceramide, C2C12 myotubes, Insulin resistance, Oxidative capacity, Cellular stress, T2DM

## Abstract

**Background:**

Chronic metabolic overload leads to insulin resistance in a variety of tissues. It has been shown that exposure to saturated fatty acid palmitate can cause insulin resistance in skeletal muscle cells. Fatty acid induced synthesis of ceramide is considered to be one of the major causes for insulin resistance. Both *de novo* synthesis and sphingomyelin hydrolysis by sphingomyelinase are implicated for ceramide generation. Aim of this study was to evaluate the impact of neutral sphingomyelinase (nSMase) inhibition on saturated fatty acid induced lipotoxicity and insulin resistance in skeletal muscle myotubes.

**Results:**

Treatment of saturated fatty acid (palmitate) but not unsaturated fatty acid (oleate) caused an up-regulation in expression of various nSMase genes which are associated with ceramide synthesis through the salvage pathway. Inhibition of nSMase by a pharmacological inhibitor (GW4869) partially reverted the palmitate induced insulin resistance in C2C12 myotubes. Inhibition of nSMase improved metabolic functions of myotubes as measured by improved oxidative capacity in terms of increased mitochondrial number, PGC1α expression and ATP levels with concomitant decrease in intramyocellular triglyceride levels. Palmitate induced inflammatory response was also reduced by nSMase inhibitor. GW4869 treatment reduced palmitate induced oxidative and endoplasmic reticulum stress and improved cell survival.

**Conclusion:**

In this study, we provide evidences that inhibition of nSMase can protect skeletal muscles from saturated fatty acid induced insulin resistance, metabolic dysfunction, cellular stress and inflammation.

**Electronic supplementary material:**

The online version of this article (doi:10.1186/2193-1801-3-255) contains supplementary material, which is available to authorized users.

## Background

Insulin resistance is a central phenomenon under metabolic disorders such as type 2 diabetes mellitus (T2DM). Understanding the defects in metabolism and underlying regulatory molecular network is of importance to design an effective therapy to manage T2DM and peripheral insulin resistance. Dealing with insulin resistance in skeletal muscle is of prime importance because this tissue accounts for as high as 95% glucose disposal during hyperglycemic hyperinsulinemic condition (Baron et al. 1988) and it is the major site for energy expenditure (van der Vusse and Reneman 1996).

Skeletal muscles from diabetic patients show impaired glucose uptake, storage and utilization because of diminished insulin signaling (Kelley et al. 1996; Kim et al. 1999; Krook et al. 2000; Bouzakri et al. 2003). Insulin resistance mediated defects are not limited only to glucose metabolism instead can influence several aspects of metabolism like overall reduction in the oxidative capacity as evident by the number/activity of mitochondria and reduced ATP levels (Simoneau and Kelley 1997; Kelley et al. 2002; Scheuermann-Freestone et al. 2003; Schrauwen and Hesselink 2004; Asmann et al. 2006; Szendroedi et al. 2007). These impairments are also accompanied by increased oxidative and endoplasmic reticulum (ER) stress and chronic low grade inflammation (Hey-Mogensen et al. 2010).

One of the major factors causing insulin resistance is elevated plasma free fatty acids (FFA) levels (Itani et al. 2002). In fact, increased rate of lipolysis from adipose tissue and VLDL secretion from liver are observed in T2DM patients and both of these might contribute for muscle FA overload. Further, augmentation in muscle fat storage is contributed by raised plasma FFA level, increased fat uptake (Bonen et al. 2004), and defective or incomplete oxidation in skeletal muscle of diabetic patients (Mootha et al. 2003; Patti et al. 2003; Mensink et al. 2007; Mogensen et al. 2007; Koves et al. 2008). Fatty acid overload leads to its accumulation in skeletal muscle mainly in form of diacylglycerol (DAG), triglyceride (TG) or ceramide in obese and T2DM patients (Goodpaster et al. 2000; Bonen et al. 2004). Increased fat storage in form of TG can be directly correlated to insulin resistance in the sedentary subjects (McGarry 2002) but not in the exercise trained ones (Goodpaster et al. 2001). On the other hand, storage of fat in terms of neutral TG might be a protective mechanism (Listenberger et al. 2003). This hypothesis is strengthened by studies which showed that unsaturated fatty acid oleate, which is a preferred substrate for TG synthesis, did not demonstrate toxic impacts (Listenberger et al. 2003; Yuzefovych et al. 2010). These evidences lead to the speculation that lipid metabolism in the form other than TG storage is required for development of insulin resistance.

Metabolism of lipid into complex lipid such as ceramide is reported to develop insulin resistance. Ceramide levels in cells are controlled by *de novo* synthesis or by its generation through salvage pathway using sphingomyelinase (SMase). The enzyme serine palmitoyl transferase (SPT) catalyzes the rate limiting step for *de novo* sphingolipid synthesis. Its expression and activity are increased in T2DM, insulin resistant subjects, and in response to saturated fatty acid and inflammatory cytokines. A number of studies indicated that pharmalogical inhibition of SPT can protect from insulin resistance and lipotoxicity (Holland et al. 2007; Ussher et al. 2010). The *de novo* synthesis of ceramides is triggered primarily from saturated fatty acids, which are one of the major contributors for insulin resistance, under various conditions relevant to metabolic syndrome such as lipotoxicity, inflammation, glucocorticoids (Holland et al. 2007; Zierath 2007; Blachnio-Zabielska et al. 2010). Treatment of cells with ceramide itself can cause insulin resistance (Schmitz-Peiffer et al. 1999; Pickersgill et al. 2007). Increased ceramide levels lead to inflammation, ER stress, oxidative stress and apoptosis (Turpin et al. 2006; de Mello et al. 2009). In obese T2DM patients, increased ceramide level is observed in plasma (Haus et al. 2009), liver (Boon et al. 2013), adipose (Blachnio-Zabielska et al. 2012) and skeletal muscle (Adams et al. 2004). Moreover, skeletal muscle ceramide levels are found to be increased in subjects at risk of development of T2DM (Straczkowski et al. 2007). In fact, there exists a positive correlation between ceramide concentration and degree of insulin resistance (Straczkowski et al. 2004,2007; Turpin et al. 2006; Haus et al. 2009; Blachnio-Zabielska et al. 2012). Therefore, it was inferred that fatty acid overload induced lipotoxicity and insulin resistance are mediated, at least in part, by synthesis of ceramide from saturated fatty acids while inhibition of ceramide synthesis could protect both cultured cells (Schmitz-Peiffer et al. 1999; Turpin et al. 2006; Pickersgill et al. 2007) and rodent model of disease (Holland et al. 2007; Yang et al. 2009; Ussher et al. 2010).

Ceramide and sphingomyelin are interconverted by enzymes sphingomyeline synthase (SMS) and sphingomyelinase (SMase). Sphingomyelin to ceramide ratio is inversely correlated to neutral SMase (nSMase) level indicating important role of nSMase in maintaining levels of these sphingolipids (Dobrzyń et al. 2002). Inflammatory cytokines can increase nSMase activity thereby increasing ceramide levels (Long and Pekala 1996). Level of nSMase is found to be increased in impaired glucose tolerant subjects (Straczkowski et al. 2007) and high fat diet induced diabetic rodents (Murase et al. 1998; Shah et al. 2008). Recent review from our laboratory exploring the gene expression profiles revealed that in T2DM patients the levels of sphingomyelinase are up-regulated (Pralhada Rao et al. 2013). Conversely, level of nSMase is decreased after exercise (Dobrzyń et al. 2002; Helge et al. 2004) leading to an increase in sphingomyelin to ceramide ratio. These reports suggest that targeting nSMase can reduce ceramide level thereby making tissue insulin sensitive. Despite these indications, there is no substantial data available in literature which can demonstrate the role of nSMase for skeletal muscle insulin resistance.

Here we report that inhibition of membrane localized neutral sphingomyelinase by a small molecule inhibitor can protect C2C12 skeletal muscle myotubes from palmitate induced insulin resistance. We demonstrate that nSMase expression is increased during palmitate treatment but not during oleate treatment. Inhibition of nSMase improves myotubes oxidative capacity and reduces triglyceride storage. Its inhibition also reduces cellular stress, inflammation and improves cell survival. Therefore, we conclude that nSMase inhibition can protect skeletal muscle from saturated FFA overload induced lipotoxic impacts. Hence, we believe that this strategy hold promise to provide opportunity for therapeutic interventions to treat muscle pathology present in metabolic syndrome.

## Results

### Palmitate treatment increases nSMase expression in C2C12 myotubes

**Figure 1:**
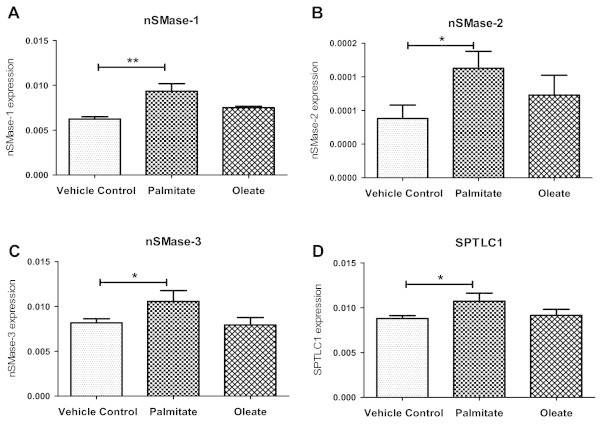
**Palmitate treatment increases nSMases expression.** Differentiated C2C12 myotubes were treated with vehicle control or palmitate or oleate (750 μM) for 16 hrs as described under ‘Methods’ section. After treatments, total RNA was isolated and mRNA levels of nSMase-1 **(A)**, nSMase-2 **(B)**, nSMase-3 **(C)** and SPTLC1 **(D)** were quantified by quantitative real time PCR using β-actin as housekeeping gene control. Data are presented as mean + standard deviation. n = 4, **P < 0.01, *P < 0.05, one way ANOVA with Newman-Keuls post test was performed for statistical analyses.

Following palmitate (750 μM) or oleate (750 μM) treatment to differentiated myotubes for 16 hrs, we measured expression of nSMase genes isoforms. Expression of nSMase-1 was up-regulated by 1.38 fold under palmitate treatment (P < 0.01, Figure 1A) when compared to vehicle control set. An increase in nSMase-2 expression was also observed under palmitate condition (1.85 fold of vehicle control, P < 0.05, Figure 1B) whereas nSMase-3 expression was slightly increased (1.29 fold of vehicle control, P < 0.05, Figure 1C). Oleate treatment was unable to induce the expression of nSMaes (Figure 1A, B, C). These results indicate an increase in nSMase expression after exposure to saturated FFA.

We next measured the expression of SPT (SPTLC1), a rate limiting enzyme in the *de novo* pathway of ceramide biosynthesis. Similar to an increase in nSMase expression, SPTLC1 expression was also up-regulated under palmitate treatment (1.22 fold of vehicle control, P < 0.05) but not under oleate treatment (Figure 1D). Thus palmitate treatment can increase both *de novo* and nSMase mediated ceramide synthesis pathways indicating a potential increase in ceramide synthesis. However, we did not measure total ceramide levels in the cells but studied functional outcomes as mentioned henceforth.

### Inhibition of nSMase reduces palmitate induced insulin resistance in C2C12 myotubes

**Figure 2:**
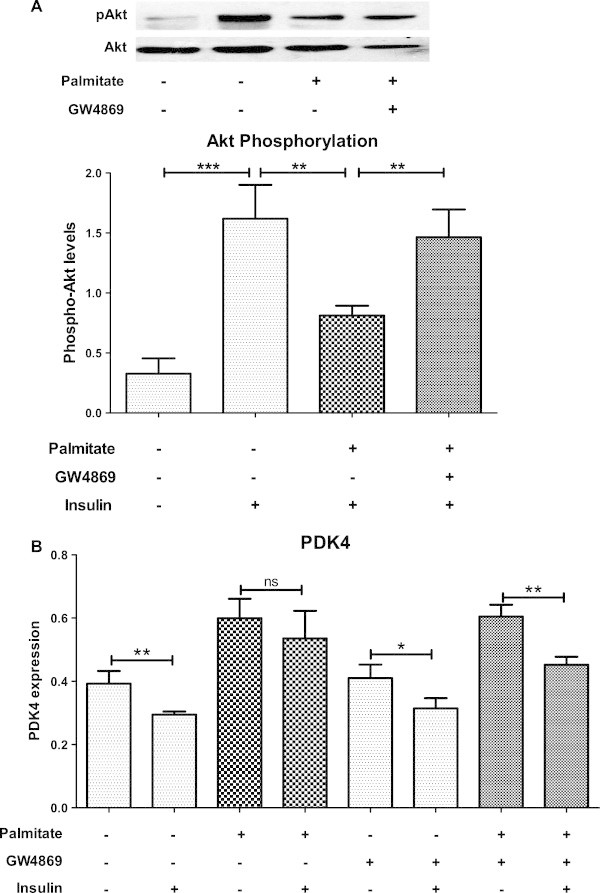
**Palmitate induced insulin resistance is alleviated by nSMase inhibition. (A)** After palmitate treatment, C2C12 myotubes were starved for 30 min. Myotubes were then treated with or without insulin for 10 min followed by lysing in lysis buffer. Insulin induced Akt phosphorylation was measured in total cell lysate by Western blotting. Unphosphorylated levels of Akt were also measured as control for phosphorylation (Upper panel). Lower panel: quantification of Akt phosphorylation (n = 6, ***P < 0.001, **P < 0.01) **(B)** C2C12 cells were treated with or without palmitate for 2 hrs in presence or absence of nSMase inhibitor (GW4869). Insulin was also present in the treatments for indicated sets. Gene expression level of PDK4 was analyzed by quantitative real time PCR using β-actin as housekeeping gene control. Data are presented as mean + standard deviation. n = 4, **P < 0.01, *P < 0.05, ns: not significant, one way ANOVA with Newman-Keuls post test was performed for statistical analyses.

We hypothesized that nSMase inhibition can reduce skeletal muscle insulin resistance caused by palmitate overload. C2C12 myotubes were treated with palmitate in presence or absence of nSMase inhibitor, GW4869 (10 μM) for 16 hrs. After 16 hrs, myotubes were treated with or without insulin for 10 min and insulin sensitivity was assessed by phosphorylated Akt. As seen in Figure 2A, insulin treatment could increase Akt phosphorylation in myotubes treated with vehicle control (P < 0.001). Palmitate treatment showed reduced Akt phosphorylation in response to insulin (P < 0.01) hence indicated development of insulin resistance. Inhibition of nSMase with palmitate treatment maintained Akt phosphorylation (P < 0.01) and thus improved insulin sensitivity (Figure 2A).

We next studied insulin induced changes in PDK4 expression, a gene whose expression level is negatively regulated with glucose oxidation. The levels of PDK4 are known to reduce with insulin treatment. Expression of PDK4 was up-regulated by palmitate exposure (Figure 2B). Insulin treatment could reduce its expression only in vehicle treated cells (0.75 fold, P < 0.01) but not in palmitate treated cells thus demonstrating progression of insulin resistance (Figure 2B). Inhibition of nSMase in this condition maintained insulin sensitivity of skeletal muscle cells as insulin was able to inhibit PDK4 expression (P < 0.01, Figure 2B). Hence we conclude that nSMase inhibition protects skeletal muscle from palmitate induced insulin resistance. Oleate treatment increased PDK4 expression in C2C12 cells, it did not cause severe insulin resistance as insulin treatment was able to inhibit its expression (P < 0.01, Additional file 1). Inhibition of nSMase maintained insulin sensitivity of oleate treated cells.

### Inhibition of nSMase results in improved oxidative capacity and metabolism

**Figure 3:**
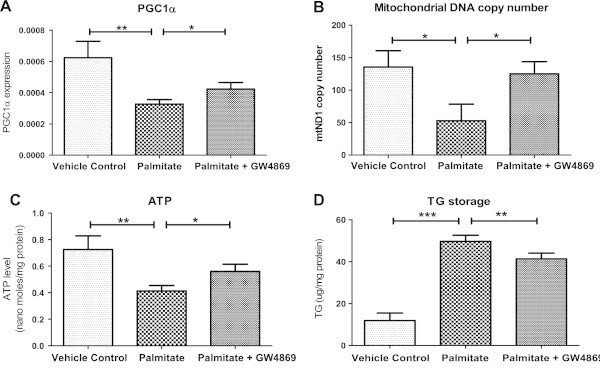
**Inhibition of nSMase improves oxidative capacity of C2C12 myotubes and reduces TG storage. (A)** Analysis of PGC1α gene expression in C2C12 myotubes cultured under palmitate condition in presence or absence of nSMase inhibitor (GW4869). β-actin was used as housekeeping gene control. **(B)** Mitochondrial DNA copy numbers were estimated by quantitative real time PCR using total DNA isolated from myotubes cultured under control or palmitate condition in presence or absence of nSMase inhibitor (GW4869). Mitochondrial DNA copy number were quantified by assessing ND1 (mitochondrial gene) level which was normalized to HPRT (nuclear gene). Cellular ATP levels **(C)** and TG storage **(D)** were quantified after culturing C2C12 myotubes under similar conditions as described under ‘Methods’ section. Data are presented as mean + standard deviation. n = 4, ***P < 0.001, **P < 0.01, *P < 0.05, one way ANOVA with Newman-Keuls post test was performed for statistical analyses.

Since insulin resistance is associated with impaired metabolism, we asked whether nSMase inhibition can improve metabolic functions of skeletal muscles. We measured impact of nSMase inhibition on expression of PGC1α, a gene which is implicated for mitochondrial biogenesis, fat oxidation and energy metabolism. Exposure of palmitate reduced PGC1α expression to 0.47 fold when compared to vehicle control (P < 0.01, Figure 3A). Inhibition of nSMase in this background increased PGC1α expression by 30% (P < 0.05, Figure 3A). Mitochondrial DNA copy number, a surrogate measurement for mitochondria number, was reduced by palmitate treatment (0.39 fold of vehicle control, P < 0.05) and nSMase inhibition could restore the mitochondrial number (P < 0.05, Figure 3B). In consistence with this, cellular ATP levels were found to be reduced under palmitate treatment (0.41 nano-moles/mg versus 0.72 nano-moles/mg under vehicle control, P < 0.01) and nSMase inhibitor increased its level by 35% (0.56 nano-moles/mg, P < 0.05, Figure 3C). Taken together, nSMase inhibition improved oxidative capacity of skeletal muscle myotubes.

We reasoned that improved insulin sensitivity and oxidative capacity by nSMase inhibition would impact fat storage. Under palmitate treatment, triglyceride storage was enhanced by 3.65 fold (50 μg/mg versus 14 μg/mg under vehicle control, P < 0.001, Figure 3D). Inhibition of nSMase under palmitate treatment led to a partial reduction in cellular triglyceride storage (41 μg/mg, P < 0.01, Figure 3C). Hence, nSMase inhibition improves overall metabolic function of skeletal muscle.

### Neutral SMase inhibition reduces inflammation and cellular stress

**Figure 4:**
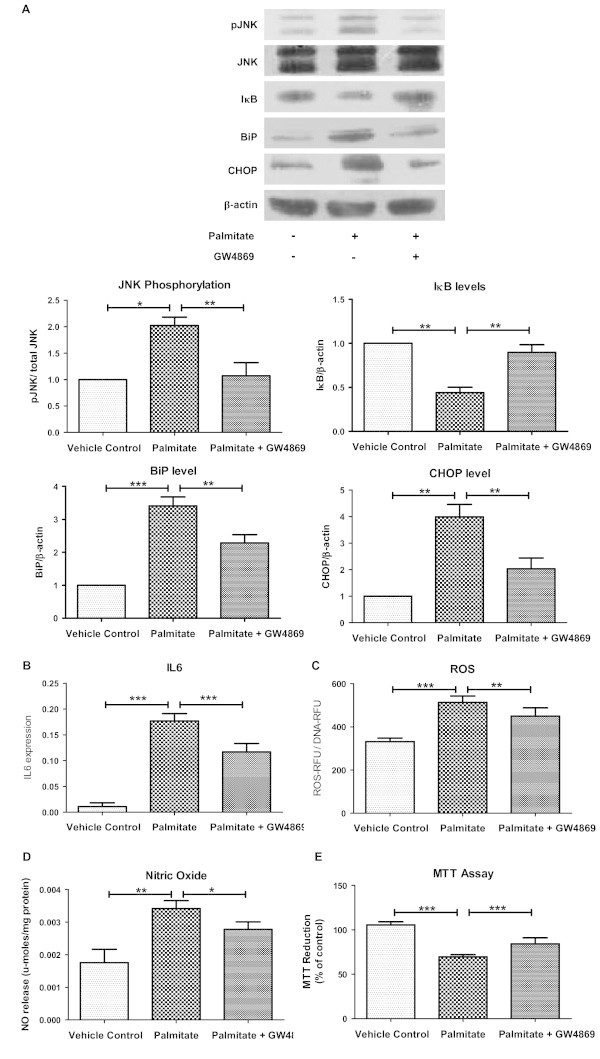
**Reduction in stress signaling, inflammation, oxidative stress and ER stress by nSMase inhibition. (A)** After palmitate treatment in presence or absence of nSMase inhibitor (GW4869), C2C12 myotubes were harvested in lysis buffer. Upper panel: Levels of phosphorylated JNK, unphosphorylated JNK, IκB, BiP and CHOP were measured using Western blotting. β-actin was used as a loading control. Lower panels: quantification of Western blotting data. **(B)** Gene expression of IL6 was measured by quantitative real time PCR using β-actin as housekeeping gene control. **(C)** After treatment, myotubes were loaded with DCFH-DA ROS indicator fluorescent probe to quantify the amount of cellular ROS. **(D)** Nitric oxide levels in culture medium were quantified using Griess reagent. **(E)** Myotubes viability was measured by MTT assay after treatment. Data are presented as mean + standard deviation. n = 4, ***P < 0.001, **P < 0.01, *P < 0.05, one way ANOVA with Newman-Keuls post test was performed for statistical analyses.

We next examined the impact of nSMase inhibition on cellular health. Palmitate treatment enhanced JNK phosphorylation (2.02 fold of control, P < 0.05) and NFκB activation as measured by reduction in IκB levels (0.44 fold of vehicle control, P < 0.01, Figure 4A) thus indicating enhanced stress signaling. Inhibition of nSMase under this background reduced stress signaling as evident by reduced JNK phosphorylation (1.07 fold of vehicle control, P < 0.01) and restoration of IκB levels (0.90 fold, P < 0.01, Figure 4A). In consistence with these data, palmitate induced increase in the IL6 expression (16 fold of control, P < 0.001) was partially decreased by nSMase inhibition (P < 0.001, Figure 4B). Treatment of myotubes with unsaturated fatty acid (oleate) was unable to increase IL6 expression and inhibition of nSMase did not change it further (Additional file 2). Thus, nSMase inhibition can reduce palmitate induced stress signaling and inflammation.

Palmitate treatment resulted in enhanced BiP (3.4 fold of control, P < 0.001) and CHOP (4 fold of control, P < 0.01) levels demonstrating increased ER stress (Figure 4A). Inhibition of nSMase reduced ER stress as measured by partial reduction in the levels of BiP and CHOP (P < 0.01, Figure 4A). Similarly, palmitate treatment elevated oxidative stress as levels of both reactive oxygen species (ROS) and reactive nitrite species (RNS) were found to be increased (Figure 4C, D). Both ROS and RNS levels were significantly reduced by nSMase inhibition (Figure 4C, D). Both ROS and RNS levels were unchanged under oleate treatment and nSMase inhibition did not show any effect under oleate (Additional file 2). Hence, palmitate induced both oxidative and ER stress are reduced by nSMase inhibitor.

Next, we analyzed the impact of palmitate treatment on myotubes survival. Palmitate treatment reduced cell survival as measured by MTT assay (70% of vehicle control, P < 0.001, Figure 4E). However, oleate treatment did not reduce cell survival (Additional file 2). Inhibition of nSMase under palmitate condition protected myotubes survival (84% of control, P < 0.001, Figure 4E). Taken together, nSMase inhibition reduces cellular stress and enhances cell survival under palmitate treatment.

## Discussion

Lipid mediators are important signaling molecules for tissue homeostasis. Ceramides are potent lipid signaling molecules and act as metabolic hub because many other sphingolipids are derived from it (Scheffer et al. 2011; Pralhada Rao et al. 2013). Ceramide biology is becoming increasingly important because ceramide can impair activation of insulin signaling pathway and hence any modulation in its content can lead to better insulin sensitivity. In fact, a direct correlation exists between the concentration of saturated fatty acid present and the amount of ceramide generated (Adams et al. 2004). Also there is a direct correlation between amount of ceramide and degree of insulin resistance (Straczkowski et al. 2004,2007; Turpin et al. 2006; Haus et al. 2009; Blachnio-Zabielska et al. 2012). Therefore, it is believed that generation of ceramide in response to FFA overload is one of the most important events causing insulin resistance (Schmitz-Peiffer et al. 1999).

FFA exposure initiates *de novo* synthesis of sphingolipids and concomitantly ceramide levels are also increased (Schmitz-Peiffer et al. 1999 Hu et al. 2009). Consequently, a number of groups tried to inhibit *de novo* synthesis of sphingolipids and reported better insulin sensitivity (Chavez et al. 2003; Powell et al. 2004; Holland et al. 2007; Watson et al. 2009; Ussher et al. 2010). However, it should be noted that not all species of sphingolipids can prompt insulin resistance. In fact, generation of sphingosine-1-phosphate (S1P) by sphingosine kinase (Hu et al. 2009) can ameliorate insulin resistance. S1P can increase glucose uptake in skeletal muscle cells (Rapizzi et al. 2009). Over-expression of sphingosine kinase to generate S1P or administration of S1P analog, FTY720, protect animal model of diabetes from insulin resistance (Kendall and Hupfeld 2008; Bruce et al. 2012,2013). Moreover, conversion of ceramide to sphingosine by ceramidase hence providing substrate for S1P generation exerts negative correlation with FFA induced insulin resistance (Chavez et al. 2003,2005). Hence, we sought to inhibit ceramide generation from sphingomyelin hydrolysis though the action of enzyme SMases which is found to be up-regulated during glucose intolerance (Murase et al. 1998; Straczkowski et al. 2007; Shah et al. 2008).

We exposed differentiated C2C12 myotubes for 16 hrs to saturated FFA palmitate which is known to increase cellular ceramide contents (Schmitz-Peiffer et al. 1999). To inhibit nSMases, we used a small molecule inhibitor, GW4869, which is reported to reduce ceramide levels (Luberto et al. 2002). Though we ourselves did not measure the total ceramide content, we observed an increase in expression of genes involved in ceramide synthesis upon palmitate treatment and estimated the functional outcomes after intervention in the ceramide synthesis pathway. To examine the relationship between nSMase and insulin resistance, we first demonstrated that the levels of all three nSMases were increased under palmitate with attendant decrease in insulin stimulated Akt/PKB phosphorylation. Thus our *in vitro* data are in agreement with what is observed in diabetic patients. Treatment with oleate did not increase the nSMases expression and it is reported that this fatty acid does not increase the ceramide content of the cells. Inhibition of nSMases, as expected, maintained insulin stimulated Akt phosphorylation indicating improved insulin sensitivity. Our data show that under nSMase inhibition, PDK4 expression was increased by palmitate and was decreased by insulin thus suggesting that metabolic flexibility of myotubes is maintained. PDK4 is a negative regulator of glucose oxidation which inhibits pyruvate entry into mitochondria for further oxidation while fat oxidation is promoted. Metabolic flexibility or ability to switch fuel utilization from fat to glucose is lost during insulin resistance since PDK4 levels are elevated (Kim et al. 2006 Tsintzas et al. 2007; Kulkarni et al. 2012). Insulin induced increase in Akt phosphorylation and decrease in PDK4 expression in myotubes cultured under nSMase inhibitor suggest functional insulin signaling cascade.

While a number of reports have authenticated connection between ceramide content and insulin resistant, we extended the study to see the effects on oxidative capacity given that overall oxidation is severely impaired in human patients (He et al. 2001; Oberbach et al. 2006; Koves et al. 2008). In order to study the impact on oxidative capacity we measured expression of PGC1α - master regulator of mitochondrial biogenesis and oxidative capacity, mitochondrial number and ATP levels. All these indicators of oxidative capacity were reduced under palmitate whereas nSMase inhibition could significantly restore their levels. Improvement in oxidative capacity by nSMase inhibition also resulted in moderate but statistically significant reduction in TG storage. Only partial restoration in oxidative capacity and reduction in TG storage by nSMase inhibition might be a result of defects caused by saturated FFA other than ceramide synthesis or contribution of *de novo* ceramide biosynthesis as we observed an increase in expression of SPT, a rate limiting enzyme of this pathway.

Both saturated FFA and ceramide activate JNK and NFκB signaling which can lead to cellular stress (Itani et al. 2002; Yuzefovych et al. 2010; MohammadTaghvaei et al. 2012). Therefore we examined JNK and NFκB activation, oxidative and ER stress and generation of inflammatory cytokines. Consistent with the data on oxidative capacity, nSMase inhibition only partially reduced cellular stress and IL6 expression again indicating about role of FFA other than ceramide synthesis for cellular stress or contribution of *de novo* ceramide biosynthesis. Importance of *de novo* ceramide biosynthesis in this regard is already reported. One possible explanation about the role of FFA other than ceramide synthesis is that extracellular FFA can directly activate toll like receptors (TLRs) which downstream activate JNK and NFκB (Senn 2006). JNK and NFκB activation results in inflammatory cytokines production and elevation of cellular stress which itself can impair oxidative capacity and insulin signaling.

Overall our data establish, for the first time, the role of nSMase in protecting skeletal muscle from saturated FFA induced lipotoxic impacts while maintaining insulin sensitivity.

## Conclusions

**Figure 5:**
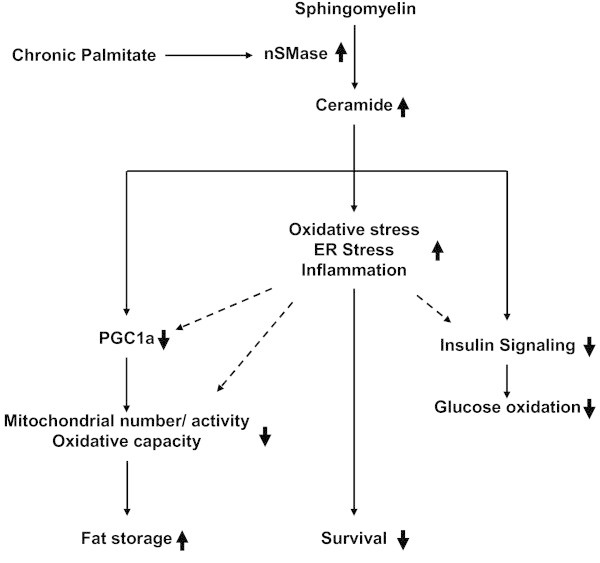
**Saturated FFA mediated impairment in skeletal muscle and its reversal by nSMase inhibition.** Sphingomyelin can be converted into ceramide by nSMases. Palmitate mediated up-regulation of nSMases can expedite this process of ceramide generation. Increased ceramide can lead to an inhibition on insulin signaling pathway thereby reducing glucose oxidation. In this condition PGC1α levels are reduced with a concomitant decrease in muscle oxidative capacity. Because of impaired oxidative function and palmitate overload, fat storage in form of TG is increased. Palmitate overload mediated increased ceramide levels elevate cellular oxidative stress, ER stress and inflammation which can severely affect cell survival. Elevated stress and inflammation can directly inhibit insulin signaling and can impair oxidative capacity (represented as dashed arrows). Palmitate mediated impact on cellular responses is represented by a thick up or down arrow placed just after particular cellular response. Inhibition of nSMase under this background reverses palmitate mediated impact on cellular response.

In this study, we report that inhibition of nSMase can prevent saturated FFA overload induced insulin resistance and multiple defects in metabolism. Inhibition of nSMase can also reduce palmitate induced stress and inflammation. Palmitate but not oleate treatment up-regulates expression of all three nSMases which generate ceramide from sphingomyelin. Palmitate treatment led to insulin resistance as well as to reduced oxidative capacity. Exposure of myotubes to palmitate caused oxidative stress, ER stress, stress signaling, inflammatory cytokine production and reduced myotubes survival. We show that these deleterious impacts of palmitate are largely by increased nSMase levels as inhibition of nSMase could revert these impacts (Figure 5). Hence, inhibition of nSMase can be a good approach to reduce skeletal muscle insulin resistance and defects in metabolism.

## Methods

### Cell culture and palmitate treatment

C2C12 myoblast cell line (ATCC) was maintained in undifferentiated state at low confluency in DMEM (Sigma) containing 25 mM glucose, 10% fetal bovine serum (Invitrogen), penicillin and streptomycin. Myoblasts were differentiated into myotubes in multi-well plates after growing till complete confluency in growth medium and followed by culturing in reduced serum (2%) medium for four days with media change at every alternate day. Fully differentiated cells evident by long multi-nucleated myotubes were used for the experiments.

Palmitate or oleate (Sigma) were dissolved in 50% ethanol followed by conjugation with 10% BSA. Differentiated myotubes received BSA conjugated palmitate or oleate (5 mM) to a final concentration of 750 μM and were cultured for 16 hrs. Vehicle control treatment included equal amount of BSA and ethanol for 16 hrs. For inhibition of nSMases, GW4869 (Sigma) was also added to a final concentration of 10 μM along with palmitate or oleate for 16 hrs.

### Analysis of gene expression

Differentiated myotubes were cultured with indicated treatment for 16 hrs. After 16 hrs of treatment, total RNA was extracted using Trizol reagent (Invitrogen) and converted into cDNA with reverse transcriptase and random hexamer primers (Applied Biosystems). A total of 5 ng of cDNA was used for real time PCR for quantification of gene expression using SYBR Green PCR Master Mix (Eurogenetic). Genes analyzed in this study were nSMase-1, nSMase-2, nSMase-3, SPTLC1, PDK4, PGC1α and IL6. β-actin was used as a housekeeping gene control.

For PDK4 (pyruvate dehydrogenase kinase 4) expression, C2C12 cells were cultured for 2 hrs in presence or absence of vehicle, palmitate (750 μM), oleate (750 μM), GW4869 (10 μM) or insulin (100 nM). Cells were then harvested for gene expression analysis.

### Western blotting

After treatment myotubes were lysed and amounts of total proteins were estimated using Bradford reagent (Bio-Rad). Equal amount of protein (30 μg) was resolved by SDS-PAGE and then transferred to nitrocellulose membrane and signals were developed by chemiluminescence. Primary antibodies used were phospho-Akt, Akt, phospho-JNK, JNK, BiP, CHOP, β-actin (Cell Signaling Technology) and IκB (Abcam).

For the estimation of insulin sensitivity by Akt phosphorylation, myotubes were cultured under vehicle control or palmitate treatment in the presence or absence of GW4869 and then incubated in serum and glucose free medium for 30 min. Subsequently, the myotubes were treated with insulin (100 nM) for 10 min and were then harvested for western blotting.

Western blot bands were quantified by densitometric analysis using Image-J software (NIH).

### Estimation of mitochondrial DNA copy number

Post 16 hrs of culture under indicated conditions, total DNA was isolated from myotubes. This total DNA (5 ng) was used for quantitative real time PCR using a primer pair specific to mitochondrial DNA (ND1) and a primer pair specific to nuclear DNA (HPRT). Mitochondrial DNA copy number was normalized to nuclear DNA.

### Estimation of cellular ATP levels

After 16 hrs of culture, myotubes were harvested for estimation of cellular ATP content using commercially available kit (ATP determination kit, Invitrogen) as per manufacturer’s protocol. Amount of ATP was normalized to total cellular protein.

### Estimation of triglyceride levels

After culture, myotubes were lysed and clear lysate was used for TG estimation using a commercially available kit (DiaSys). TG levels were normalized to cellular proteins measured using Bradford reagent (Bio-Rad).

### Estimation of ROS

After treatments for 16 hrs, myotubes were loaded with DCFH-DA dye (Invitrogen) for 1 h to measure ROS levels. Amount of ROS was normalized to total cellular DNA which was measured using bis-benzamide (Sigma). Bis-benzamide was added for 15 min. Myotubes were lysed and lysates were transferred to 96-well black well plate. DCF fluorescence was measured at 485 nm excitation and 528 nm emission. DNA levels were measured at 360 nm excitation and 460 nm emission. Data are represented as ROS fluorescence (RFU)/DNA fluorescence (RFU).

### Estimation of nitric oxide release

Nitric oxide release was measured in cell culture medium. After treatment, culture medium was centrifuged to remove any debris. Culture medium was mixed with equal amount of Griess reagent (Sigma) and incubated for 15 min followed by absorption measurement at 540 nm. Amount of nitric oxide release was quantified by a standard curve prepared using sodium nitrite and were normalized to total cellular proteins measured using Bradford reagent (Bio-Rad).

### MTT assay

After treatment, MTT dye was added to myotubes and incubated for 3h. After incubation culture medium was discarded and DMSO was added to each well to dissolve the reduced form of MTT (formazan). Amount of MTT reduced was estimated by 560 nm absorbance after correction with 640 nm absorbance. Reduction of MTT into formazan was represented as % of vehicle control.

### Statistical analysis

Data are represented as mean with standard deviation or mean with standard error of mean. Statistical significances were calculated by one way ANOVA with Newman-Keuls post test using GraphPad Prism. P-values were calculated between groups as indicated in figures and were represented as *P < 0.05, **P < 0.01 and ***P < 0.001.

## Electronic supplementary material

Additional file 1: **Impact of oleate treatment, nSMase inhibition and insulin on PDK4 expression.** C2C12 cells were treated with or without oleate for 2 hrs in presence or absence of nSMase inhibitor (GW4869). Insulin was also present in the treatments for indicated sets. Gene expression level of PDK4 was analyzed by quantitative real time PCR using β-actin as housekeeping gene control. In vehicle control treated cells, insulin significantly inhibited PDK4 expression. Though oleate treatment increased the PDK4 expression, it did not cause insulin resistance as insulin was able to reduce the expression of PDK4. Inhibition of nSMase by GW4869 maintained the insulin sensitivity of C2C12 cells. Data are presented as mean + standard deviation. n = 4, **P < 0.01, *P < 0.05, one way ANOVA with Newman-Keuls post test was performed for statistical analyses. (DOC 22 KB)

Additional file 2: **Oleate treatment does not show any impact on inflammation, oxidative stress and cell survival.** Myotubes were treated for 16 hrs either with vehicle control or with oleate (750 μM) in presence or absence of GW4869. Gene expression of IL6 was measured by quantitative real time PCR using β-actin as housekeeping gene control (A). After treatment, myotubes were loaded with DCFH-DA ROS indicator fluorescent probe to quantify the amount of cellular ROS (B) and nitric oxide levels in culture medium were quantified using Griess reagent (C). Myotubes viability was measured by MTT assay (D). Data are presented as mean + standard deviation. n = 4, one way ANOVA with Newman-Keuls post test was performed for statistical analyses and no statistical significance was observed among treatments indicating oleate treatment did not cause inflammation and cellular stress and did not impact viability. (DOC 526 KB)

## Abbreviations

SMase: Sphingomyelinase
nSMase: Neutral SMase
SPT: Serine palmitoyl transferase
S1P: Sphingosine-1-phosphate
T2DM: Type-II Diabetes mellitus
FFA: Free fatty acid
TG: Triglyceride
ER: Endoplasmic reticulum
PDK4: Pyruvate dehydrogenase kinase 4
PGC1α: Peroxisome proliferator-activated receptor gamma, Coactivator 1 Alpha
IL6: Interleukin 6
ROS: Reactive oxygen species.
