# Supplementary material for: Inhibition of neutral sphingomyelinases in skeletal muscle attenuates fatty-acid induced defects in metabolism and stress
Source: Springerplus. 2014 May 20;3:255. doi: 10.1186/2193-1801-3-255 (PMC4039661; doi:10.1186/2193-1801-3-255)
Supplement: Supplementary file 1 — Additional file 1: Impact of oleate treatment, nSMase inhibition and insulin on PDK4 expression. C2C12 cells were treated with or without oleate for 2 hrs in presence or absence of nSMase inhibitor (GW4869). Insulin was also present in the treatments for indicated sets. Gene expression level of PDK4 was analyzed by quantitative real time PCR using β-actin as housekeeping gene control. In vehicle control treated cells, insulin significantly inhibited PDK4 expression. Though oleate treatment increased the PDK4 expression, it did not cause insulin resistance as insulin was able to reduce the expression of PDK4. Inhibition of nSMase by GW4869 maintained the insulin sensitivity of C2C12 cells. Data are presented as mean + standard deviation. n = 4, **P < 0.01, *P < 0.05, one way ANOVA with Newman-Keuls post test was performed for statistical analyses. (DOC 22 KB) [file 40064_2014_973_MOESM1_ESM.doc]

**Additional file-1. Impact of oleate treatment, nSMase inhibition and insulin on PDK4 expression**

C2C12 cells were treated with or without oleate for 2 hrs in presence or absence of nSMase inhibitor (GW4869). Insulin was also present in the treatments for indicated sets. Gene expression level of PDK4 was analyzed by quantitative real time PCR using β-actin as housekeeping gene control. In vehicle control treated cells, insulin significantly inhibited PDK4 expression. Though oleate treatment increased the PDK4 expression, it did not cause insulin resistance as insulin was able to reduce the expression of PDK4. Inhibition of nSMase by GW4869 maintained the insulin sensitivity of C2C12 cells. Data are presented as mean + standard deviation. n=4, **P<0.01, *P<0.05, one way ANOVA with Newman-Keuls post test was performed for statistical analyses.
