# Supplementary material for: Inhibition of neutral sphingomyelinases in skeletal muscle attenuates fatty-acid induced defects in metabolism and stress
Source: Springerplus. 2014 May 20;3:255. doi: 10.1186/2193-1801-3-255 (PMC4039661; doi:10.1186/2193-1801-3-255)
Supplement: Supplementary file 2 — Additional file 2: Oleate treatment does not show any impact on inflammation, oxidative stress and cell survival. Myotubes were treated for 16 hrs either with vehicle control or with oleate (750 μM) in presence or absence of GW4869. Gene expression of IL6 was measured by quantitative real time PCR using β-actin as housekeeping gene control (A). After treatment, myotubes were loaded with DCFH-DA ROS indicator fluorescent probe to quantify the amount of cellular ROS (B) and nitric oxide levels in culture medium were quantified using Griess reagent (C). Myotubes viability was measured by MTT assay (D). Data are presented as mean + standard deviation. n = 4, one way ANOVA with Newman-Keuls post test was performed for statistical analyses and no statistical significance was observed among treatments indicating oleate treatment did not cause inflammation and cellular stress and did not impact viability. (DOC 526 KB) [file 40064_2014_973_MOESM2_ESM.doc]

**Additional file-2. Oleate treatment does not show any impact on inflammation, oxidative stress and cell survival**

Myotubes were treated for 16 hrs either with vehicle control or with oleate (750μM) in presence or absence of GW4869. Gene expression of IL6 was measured by quantitative real time PCR using β-actin as housekeeping gene control **(A)**. After treatment, myotubes were loaded with DCFH-DA ROS indicator fluorescent probe to quantify the amount of cellular ROS **(B)** and nitric oxide levels in culture medium were quantified using Griess reagent **(C)**. Myotubes viability was measured by MTT assay **(D)**. Data are presented as mean + standard deviation. n=4, one way ANOVA with Newman-Keuls post test was performed for statistical analyses and no statistical significance was observed among treatments indicating oleate treatment did not cause inflammation and cellular stress and did not impact viability.
